# Supplementary material for: Apigenin Ameliorates H2O2-Induced Oxidative Damage in Melanocytes through Nuclear Factor-E2-Related Factor 2 (Nrf2) and Phosphatidylinositol 3-Kinase (PI3K)/Protein Kinase B (Akt)/Mammalian Target of Rapamycin (mTOR) Pathways and Reducing the Generation of Reactive Oxygen Species (ROS) in Zebrafish
Source: Pharmaceuticals (Basel). 2024 Sep 30;17(10):1302. doi: 10.3390/ph17101302 (PMC11510047; doi:10.3390/ph17101302)
Supplement: Supplementary file 1 [file pharmaceuticals-17-01302-s001.zip › Supplementary material.pdf]

Figure S1.

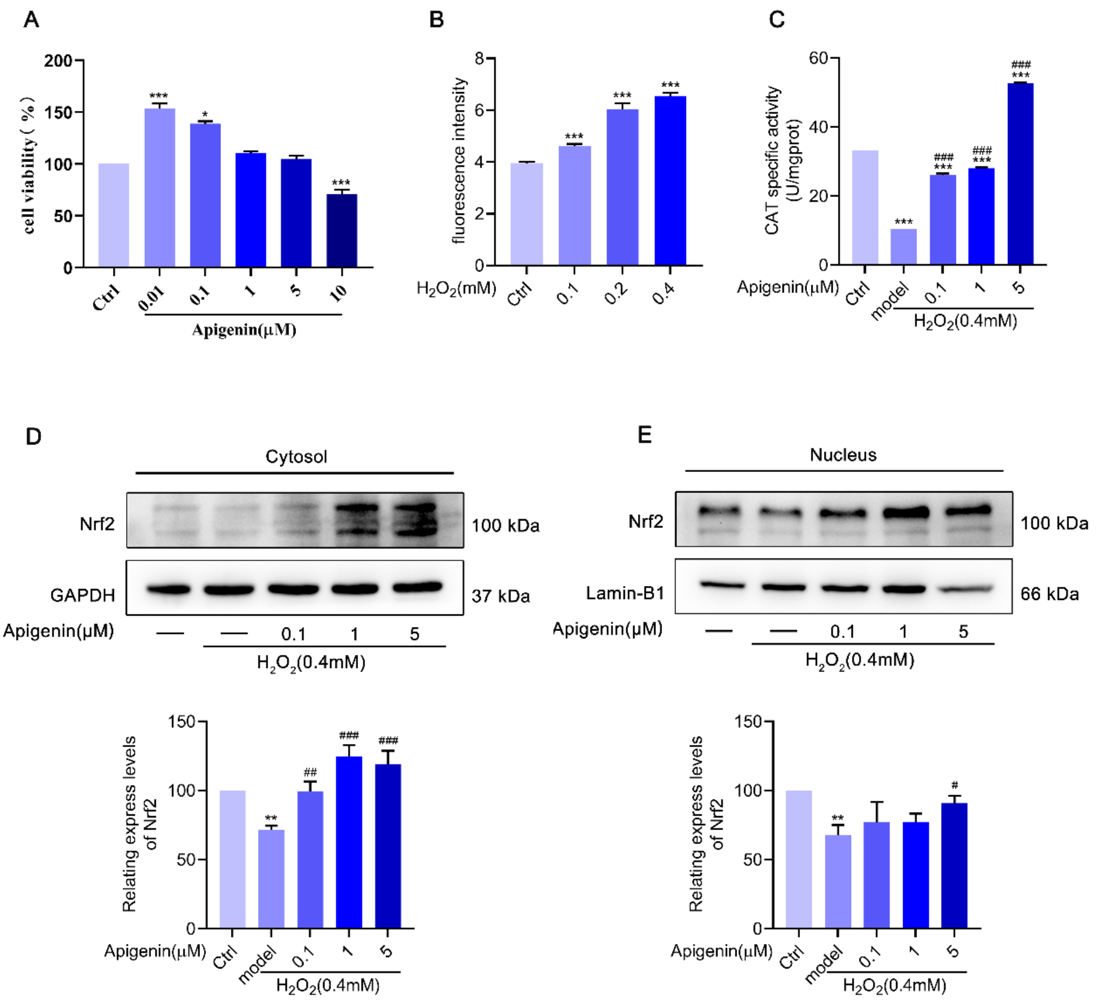

Figure S1 Captions

Fig. S1A Effect of apigenin on cell viability of B16F10 cells. The data was presented as the mean ± SEM, n ≥ 3, **p* < 0.05, ****p*< 0.001, vs control. **(S1B)** Effect of H_2O_2 on ROS levels of B16F10 cells (n=3). Data are expressed as the mean ± SEM (n = 3). ****p* < 0.001 *vs.* control. **(S1C)** Effects of apigenin on CAT activity in oxidant model of B16F10 cells. The data were presented as the mean ± SEM, n ≥ 3, ****p* < 0.001, vs control. $^{###}p < 0.001$, vs model. **(S1D) and (S1E)** Effect of apigenin on Nrf2 expression in the nucleus and cytosol of B16F10 cells under oxidative stress state. The data were presented as the mean ± SEM, n ≥ 3, ***p* < 0.01, vs control. $^{#}p$ <

0.05 $^{\#\#}p < 0.01$, $^{\#\#\#}p < 0.001$, vs model.
